# Concurrent head and neck paragangliomas and pulmonary carcinoid tumor: A rare clinical entity

**DOI:** 10.1016/j.radcr.2025.10.029

**Published:** 2025-11-11

**Authors:** Faria Nisar, Hoo Jung Rhim, Roman Finocchiaro, Vikas Jain

**Affiliations:** a The Department of Anesthesiology and Pain Management, The MetroHealth System, Cleveland, OH 44109, USA; b The Department of Radiology, The MetroHealth System, Cleveland, OH 44109, USA

**Keywords:** Paraganglioma, Multiple paragangliomas, Neuroendocrine tumors, Pulmonary carcinoid tumor

## Abstract

We report the first documented case of a 61-year-old female presenting with concurrent multiple head and neck paragangliomas (HNPGLs) and a pulmonary carcinoid tumor, representing an unprecedented association of synchronous neuroendocrine tumors (NETs). This rare coexistence presented unique diagnostic challenges requiring multimodal imaging, including FDG PET-CT, contrast-enhanced CT, MRI, and Cu-64 DOTATATE PET-CT for comprehensive tumor characterization. Management employed a tailored multidisciplinary approach: surgical resection of the dominant carotid body tumor with excellent local control, stereotactic radiation therapy for remaining cervical lesions (45 Gy), and active surveillance with octreotide therapy for the pulmonary carcinoid. At 24-month follow-up, all lesions remained stable with no evidence of progression or new neurological deficits. Despite declining genetic testing, the patient's multiple paragangliomas strongly suggest hereditary predisposition, emphasizing the critical importance of genetic counseling in such presentations. The successful management approach provides a framework for similar complex presentations and highlights the potential for shared molecular mechanisms underlying synchronous neuroendocrine tumorigenesis.

## Introduction

Paragangliomas (PGLs) represent a group of neuroendocrine tumors (NETs) that originate from neural crest-derived tissue [1,2]. Paragangliomas develop in the parasympathetic or sympathetic ganglia located outside the adrenal medulla [3]. head and neck paragangliomas (HNPGLs) comprise 65%-70% of paragangliomas but only 0.6% of head and neck tumors; 6%-19% of paragangliomas are metastatic, adversely affecting survival [3].

HNPGLs can be classified into 3 primary categories based on their anatomical location: carotid body tumors (CBTs) (60%); jugulotympanic paragangliomas (JTPGs), (35%); and vagal paragangliomas (VPGs), (5%) [4]. These highly vascular tumors usually present as slow-growing, painless masses, often clinically silent until later symptoms from mass effect or catecholamine secretion [5]. The evaluation of paragangliomas requires both structural imaging modalities, including computed tomography (CT) and magnetic resonance imaging (MRI), and functional imaging with nuclear medicine studies with variable radiotracers [6].

The presence of multiple PGLs, as observed in our patient, occurs in approximately 10% of sporadic cases and up to 40% of familial tumors [7]. These presentations often warrant comprehensive clinical, radiological, and genetic evaluation to rule out hereditary syndromes and guide appropriate management strategies. A particularly rare clinical entity is the concurrent presence of multiple PGLs and pulmonary carcinoid tumors. While both tumor types are of neuroendocrine origin, their coexistence is extremely uncommon in medical literature and may suggest shared pathogenic mechanisms or genetic susceptibility.

## Case presentation

**Fig. 1:**
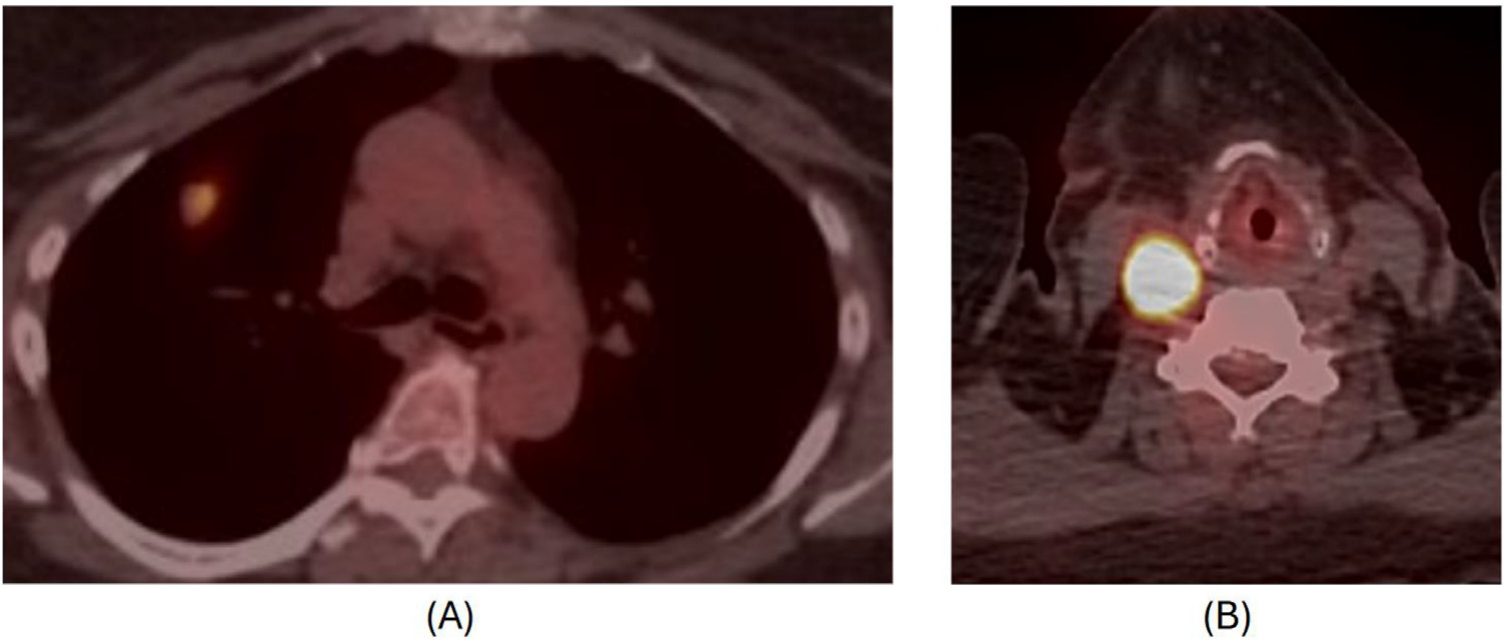
(A, B): Initial PET CT axial image demonstrating hypermetabolic solid lung nodule measuring up to 1.7 cm (A). Hypermetabolic mass was incidentally identified near the right carotid bifurcation (B).

A 61-year-old female with a history of hypertension, anxiety, palpitation, and previously identified pulmonary nodules underwent surveillance PET-CT 5 years later, which incidentally revealed an FDG-avid mass in the neck and a solid lung nodule in the right upper lobe measuring 1.4 × 1.7 cm with increased FDG uptake (Fig. 1). Her cardiac workup revealed a high systolic blood pressure of >180 mmHg and a sinus rhythm with a heart rate of 50-60 bpm at the time of palpitations. Plasma and urinary catecholamines (Epinephrine, Norepinephrine, Dopamine) and 24-hour urinary vanillylmandelic acid (VMA) levels (reference range: < or = 6.0 mg/24hr) were within normal limits.

**Fig. 2:**
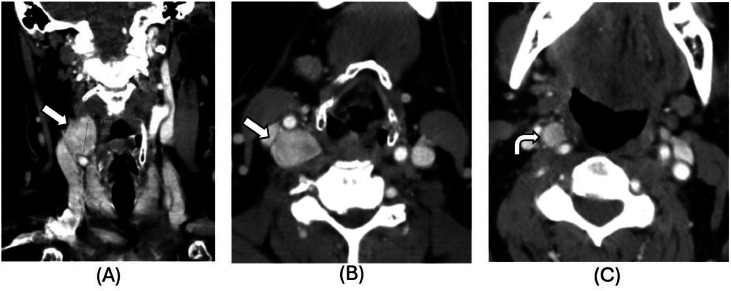
(A-C): Follow-up coronal (A) and axial (B, C) CT neck images demonstrating 2 distinct hyperdense masses, one inferior to the right carotid bifurcation near the posterior margin of the common carotid artery (white arrows, A-B) and the smaller lesion splaying the carotid bifurcation (curved arrow, C). Given the classical findings of carotid body tumor/paraganglioma, nuclear medicine Cu-64 Dotate PET/CT imaging was recommended, although metastatic adenopathy was still on the differential.

**Fig. 3:**
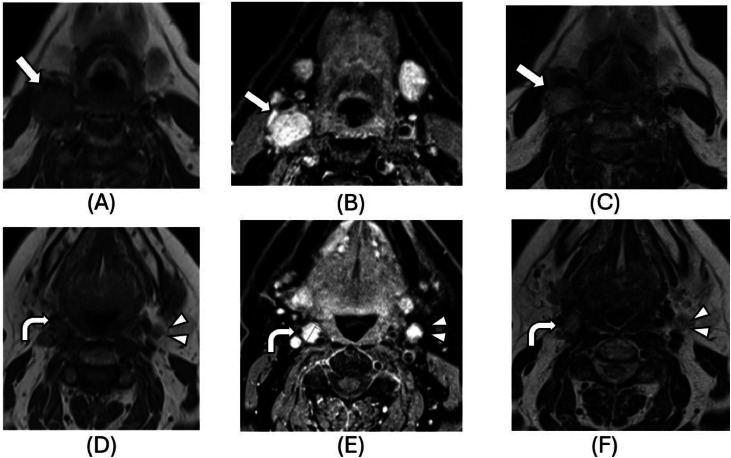
(A-F): Magnetic resonance imaging of the neck in April 2023. Axial T1WI pre-contrast (A, D) and post-contrast (B, E), and axial T2WI (C, F) images below the level of carotid bifurcation (A, B, C, F) and at the level of carotid bifurcation (D, E) demonstrating homogeneously enhancing soft tissue mass (arrows, A-C; curved arrows, D-F), of which demonstrate T2 hyperintensity and contrast enhancement in keeping with classical imaging findings of paraganglioma. Similar rounded nodules in the left level IIA station (arrowheads, D-F) and near the dorsal margin of the right carotid sheath at C1-C2 (not shown) are suspicious for a similar etiology.

Contrast-enhanced neck CT revealed multiple PGLs: a 1.1 × 1.0 cm carotid body tumor splaying the right carotid bifurcation, a 1.6 × 1.5 cm hyperdense mass at the posterior margin of the right common carotid artery, and a 0.6 cm vascular nodule along the internal carotid artery near the skull base (Fig. 2). MRI confirmed these findings and identified additional suspicious lesions (Fig. 3).

The patient underwent surgical resection of the right carotid body tumor followed by radiation therapy to the right neck, delivered with intensity modulated radiation therapy (IMRT) implemented with volumetric modulated arc therapy (VMAT) to reduce dose to the adjacent tissue. Delivered total dose was 45 Gy. Surgical resections of the dominant hyper-enhancing ovoid mass inferior to the carotid body tumor and the smaller peripherally enhancing mass positioned between the internal carotid and internal jugular vein were discouraged due to concerns about vagus nerve injury and associated voice changes. Both lesions were deemed inoperable and placed under radiation therapy. The patient declined genetic testing, limiting assessment of hereditary versus sporadic disease.

**Fig. 4:**
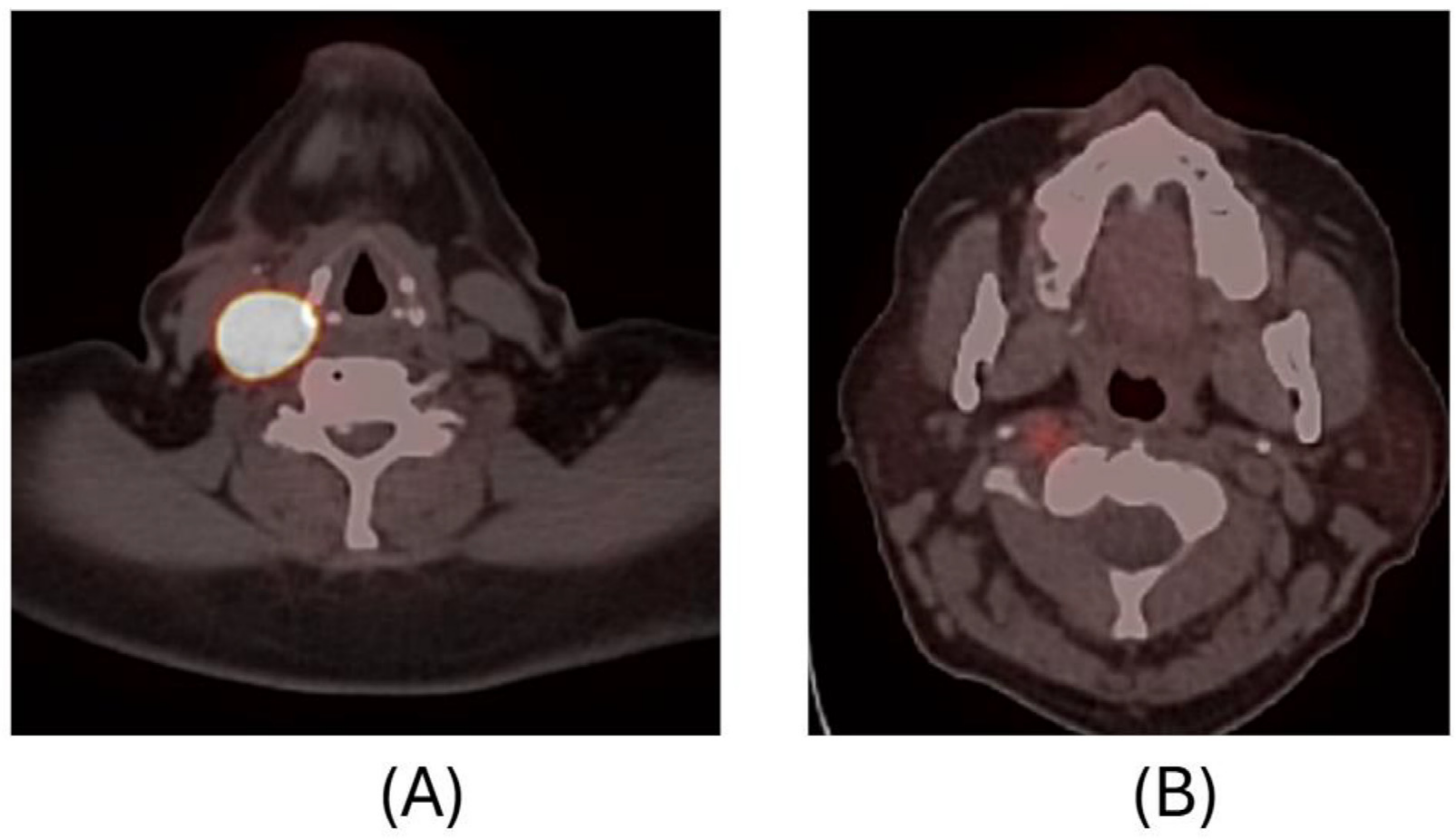
(A, B): Cu-64 Dotatate neuroendocrine PET study after surgical excision of the tumor at the right common carotid artery bifurcation with subsequent biopsy-proven diagnosis of carotid body tumor. Intense radiotracer uptake in the soft tissue nodule slightly inferior-posterior to the surgical bed, corresponding to non-resected paraganglioma shown in Figure 2 images A and B (A). A focus of mild radiotracer uptake at C1-2 level (B) likely corresponding to the 5 mm enhancing nodule along the right ICA seen on prior MR scan (not shown).

Given the multifocal nature of the neck masses and paragangliomas, nuclear medicine Cu-64 Dotatate PET/CT imaging was recommended, although metastatic adenopathy remained in the differential. Dotatate PET revealed PGLs along the right ICA and left common carotid artery (Fig. 4).

Longitudinal surveillance of the pulmonary nodules revealed slow growth of the main right upper lobe nodule. CT-guided biopsy of the lesion was performed for further investigation, and it confirmed a typical carcinoid tumor. Intramuscular octreotide was initiated. Sequential follow-up imaging showed stable cervical paragangliomas and pulmonary carcinoids.

## Discussion

### Synchronous paragangliomas and pulmonary carcinoid: a rare association

The concurrent presence of multiple head and neck paragangliomas and a pulmonary carcinoid tumor seen in the patient represents an exceptionally rare clinical entity with no previously reported cases in the literature. While both tumor types exhibit neuroendocrine origin, their simultaneous occurrence suggests potentially shared molecular pathway.

These tumors can arise from either germline variations in susceptibility genes or de novo somatic mutations. Over 20 genes have been implicated in their development, with succinate dehydrogenase (SDH) mutations being the most common genetic alteration [[2], [3], [4],^8^]. Previous studies have established that certain NETs can coexist through common genetic mechanisms [9]. For instance, VHL disease and SDH-related syndromes both predispose patients to multiple NETs through shared HIF pathways, with VHL patients developing pheochromocytomas and pancreatic NETs [10]. The Stratakis-Carney Dyad further demonstrates this concept, associating SDHx mutations with both paragangliomas and gastrointestinal stromal tumors [11]. Additionally, the discovery of SDH-deficient pancreatic NETs has expanded this spectrum, reinforcing that single genetic pathways can give rise to distinct but related tumor types [12].

### Diagnostic approach to paragangliomas

A comprehensive diagnostic approach is essential for evaluating masses suspicious of PGL, particularly in the head and neck. Although up to 95% of HNPGLs are non-secretory, biochemical testing is recommended to rule out catecholamine production, as some patients may present with hypertension, headache, palpitations, or anxiety [3].

The workup typically includes measuring plasma-free metanephrines or collecting 24-hour urine for fractionated metanephrines and catecholamines [3]. Fine needle aspiration or incisional biopsy is usually deferred until biochemical assessment is performed due to the risk of catecholamine crisis [13].

CT and MRI are critical for characterizing tumor extent and relationships to surrounding structures. Carotid body tumor typically demonstrates splaying of the internal and external carotid arteries, while jugulotympanic PGL involves the skull base and may extend to middle ear cavity. Vagal PGL usually displaces the internal carotid artery anteromedially and the internal jugular vein posterolaterally. CT is ideal for assessing osseous involvement, and contrast-enhanced scans demonstrate hypervascular masses with strong enhancement. MRI shows low T1, intermediate–high T2 signal, and intense gadolinium enhancement, with flow voids producing the classic "salt-and-pepper" appearance. Staging systems like Browne assess jugular foramen involvement and predict surgical complexity. Advanced imaging such as magnetic resonance angiography and perfusion MRI can provide additional information on vascularization and hemodynamics [4,14].

Functional imaging complements anatomical studies by targeting specific physiological processes and receptor expression patterns. The Endocrine Society recommends 18F-fluorohydroxyphenylalanine PET-CT as the preferred approach [3]. While iodine-123/131-metaiodobenzylguanidine (MIBG) can serve as an alternative option, gallium-68 (68Ga)-DOTA-somatostatin analogs demonstrate superior sensitivity in detecting HNPGLs across various genetic backgrounds. For assessing potential metastases, 18F-fluorodeoxyglucose PET/CT provides valuable information by identifying metabolically active disease sites. Nuclear medicine not only serves a diagnostic purpose but also can be expanded to a treatment modality with radionuclide therapy. Commonly used therapeutic radiopharmaceuticals include 90Y/177Lu-DOTA-somatostatin analog or 131I-MIBG [2,15]. Sensitivity may vary with genetic mutations: 68Ga-DOTATATE PET/CT is most sensitive for SDHx-related PGLs, 18F-FDOPA is preferred for VHL, RET, NF1, HIF2A, or EGLN1 mutations, and MAX-associated tumors show reduced uptake on 18F-FDG and 68Ga-DOTA tracers [2].

Patients managed with observation require serial imaging every 6-12 months. 68Ga-DOTA PET/CT facilitates the detection of vagal PGLs, and MR spectroscopy may detect SDHx mutations through succinate accumulation, although bone and air artifacts can limit practicality [16]. Screening is recommended for young patients with hypertension, treatment-resistant hypertension, episodic symptoms, relevant syndromes, family history, or suspicious adrenal incidentalomas. Biochemical testing remains essential for all HNPGL cases due to a higher malignancy risk in secretory tumors [9].

This case exemplifies the essential role of comprehensive imaging in NET evaluation. In this patient, FDG PET-CT incidentally identified both cervical and pulmonary lesions, underscoring the value of whole-body imaging in NET evaluation. Imaging findings were classic: hypervascular masses with contrast-enhanced CT enhancement, and avid Cu-64 DOTATATE uptake indicating high somatostatin receptor expression. The carotid body tumor showed characteristic splaying of the carotid arteries, aiding differentiation from other cervical masses. Multitracer PET provided superior sensitivity, with DOTATATE uptake suggesting potential SDH pathway involvement, although genetic confirmation was declined.

### Management challenges in multiple paragangliomas

Multiple PGLs pose unique challenges due to their variable sites, secretory potential, and strong genetic associations, particularly with SDH mutations [[2], [3], [4], [5],^8^,^9^,^16^,^17^]. Their proximity to critical neurovascular structures (eg, carotid artery, jugular vein, cranial nerves) can contribute to neurological deficits at presentation [18].

### Treatment considerations

HNPGLs require individualized treatment strategies ranging from observation and surveillance imaging to surgical resection and systemic therapy. Benign HNPGLs have favorable 5-year survival (up to 91%), but rates decline with metastasis: 76.8% with regional and 11.8% with distant disease. Small, asymptomatic tumors may be monitored with MRI every 6-12 months, though 13%-33% of patients may develop new cranial nerve deficits within 5 years [[3], [4], [5]].

### Surgical management

Surgery remains the only curative option when complete resection is feasible. Tumor size is closely related to operability and surgical prognosis. Shamblin classification aids operative planning for CBTs by assessing carotid involvement [5,19]. Surgical approaches are tailored to tumor type, location, and invasion, prioritizing cranial nerve preservation. According to Trache et. al., CBTs, which are partially surrounding the carotid artery, were reported to achieve >90% tumor control, although 14% of patients developed postsurgical cranial nerve deficits. Vagal PGLs often require vagus nerve transection, resulting in significant speech and swallowing complications and additional lower cranial nerve injury in 21%-39% of cases [5]. Preoperative embolization may reduce blood loss, and balloon test occlusion is recommended when carotid arterial injury is anticipated [4,5,7,16].

### Radiation therapy

Radiation therapy is a key alternative to surgery in patients with advanced age, comorbidities, bilateral disease, or swallowing dysfunction. Conventional regimen delivers 45 Gy over 5 weeks, while stereotactic radiosurgery (SRS) and stereotactic body radiation therapy (SBRT) offer more targeted approaches [16,20], achieving similar tumor control (92% vs 78%) but with fewer complications (11% vs 28%) [20].

SRS, including CyberKnife and hypofractionated approaches, allows precise treatment of larger or skull base lesions, with generally self-limited side effects such as neck edema and mucositis [20,21].

### Systemic therapy for metastatic disease

For metastatic PGLs, systemic therapy options have expanded. In 2018, the U.S. Food and Drug Administration approved iodine-131-MIBG therapy for iobenguane scan-positive, unresectable, or metastatic tumors, showing a 22% overall response rate, with 53% of responses lasting ≥6 months [3]. Additional therapies include temozolomide, thalidomide, 17-alkylamine protein inhibitors, mTOR and tyrosine-kinase inhibitors, antiangiogenic agents, Lutetium-octreotate, gene therapy, and peptide receptor radionuclide therapy with 90Y- or 177Lu-DOTA somatostatin analogs, which have shown palliative efficacy [9].

### Clinical implications and future directions

This case underscores several critical clinical principles for managing concurrent NETs. First, comprehensive whole-body imaging is essential when any NET is identified, as synchronous tumors may occur in anatomically disparate locations. Multimodal imaging approach encompassing anatomical imaging as well as functional imaging is necessary for accurate diagnosis and treatment planning.

Second, concurrent NETs require individualized, multidisciplinary management strategies with various considerations including but not limited to tumor biology, anatomical location, and patient factors. Hybrid approach combining surgery, radiation, and surveillance achieved disease control while preserving neurological function in the discussed case, thus providing a framework for similar complex presentations.

Third, genetic counseling and testing should be strongly recommended in patients with multiple NETs. High likelihood of hereditary predisposition has significant implications for surveillance, treatment selection, and family screening that extend beyond the individual patient.

Finally, long-term surveillance protocols must be established for concurrent NETs, incorporating both anatomical and functional imaging to monitor treatment response and detect progression across multiple tumor sites. Our 24-month follow-up demonstrated disease stability.

This unprecedented case warrants further investigation into shared molecular mechanisms underlying synchronous neuroendocrine tumorigenesis, and the need to further assess synchronous or metachronous NETs. Recognition of potential underlying etiology may lead to breakthroughs in surveillance strategies and individualized treatment approaches.

## Conclusion

This case demonstrates the critical importance of comprehensive, multimodal imaging in managing patients with concurrent NETs. The rare coexistence of multiple paragangliomas and pulmonary carcinoid tumors highlights the value of systematic radiological evaluation, genetic consideration, and coordinated multidisciplinary care in optimizing patient outcomes. Further studies are needed to clarify potential shared mechanisms and guide evaluation protocols for multifocal NETs.

## Patient consent

The patient did not provide written informed consent for this case report. However, all potentially identifiable information has been thoroughly removed in accordance with our institutional privacy policies. This de-identification was reviewed and confirmed in collaboration with our hospital's privacy officer to ensure compliance with HIPAA and ethical publication standards.
